# Subunit Interface Residues F129 and H294 of Human RAD51 Are Essential for Recombinase Function

**DOI:** 10.1371/journal.pone.0023071

**Published:** 2011-08-12

**Authors:** Ravindra Amunugama, Richard Fishel

**Affiliations:** 1 Biophysics Graduate Program, The Ohio State University Medical Center and Comprehensive Cancer Center, Columbus, Ohio, United States of America; 2 Department of Molecular Virology, Immunology, and Medical Genetics, Human Cancer Genetics, The Ohio State University Medical Center and Comprehensive Cancer Center, Columbus, Ohio, United States of America; 3 Physics Department, The Ohio State University Columbus, Columbus, Ohio, United States of America; Tulane University Health Sciences Center, United States of America

## Abstract

RAD51 mediated homologous recombinational repair (HRR) of DNA double-strand breaks (DSBs) is essential to maintain genomic integrity. RAD51 forms a nucleoprotein filament (NPF) that catalyzes the fundamental homologous pairing and strand exchange reaction (recombinase) required for HRR. Based on structural and functional homology with archaeal and yeast RAD51, we have identified the human RAD51 (HsRAD51) subunit interface residues HsRad51(F129) in the Walker A box and HsRad51(H294) in the L2 ssDNA binding region as potentially important participants in salt-induced conformational transitions essential for recombinase activity. We demonstrate that the HsRad51(F129V) and HsRad51(H294V) substitution mutations reduce DNA dependent ATPase activity and are largely defective in the formation of a functional NPF, which ultimately eliminates recombinase catalytic functions. Our data are consistent with the conclusion that the HsRAD51(F129) and HsRAD51(H294) residues are important participants in the cation-induced allosteric activation of HsRAD51.

## Introduction

Failure to repair DNA double strand breaks (DSBs) leads to tumorigenesis and genomic instability. Homologous recombination (HR) is an evolutionary conserved repair pathway utilized to restore DSBs. HR mediated DSB repair is initiated by resection of the 5′-end of the break to leave a 3′-single-stranded DNA (ssDNA) overhang [1], [2]. In eukaryotes, RAD51 forms a nucleoprotein filament (NPF) on the newly formed ssDNA region aided by other recombination mediators such as RAD52 in yeast and BRCA2 in vertebrates [3], [4], [5], [6], [7], [8]. The key function of the RAD51 NPF is to catalyze the homology search and initiate strand exchange. Deletion of Rad51 in mice results in embryonic lethality, while RAD51 knock down in chicken DT40 cell lines results in increased chromosomal instability [9], [10]. Even though, no mutations of RAD51 have been found in cancers, its expression is elevated in many cancer cell lines; perhaps to provide an advantage to rapidly dividing cells by repairing DSBs that would lead to replication fork collapse [11],[12],[13].

**Figure 1:**
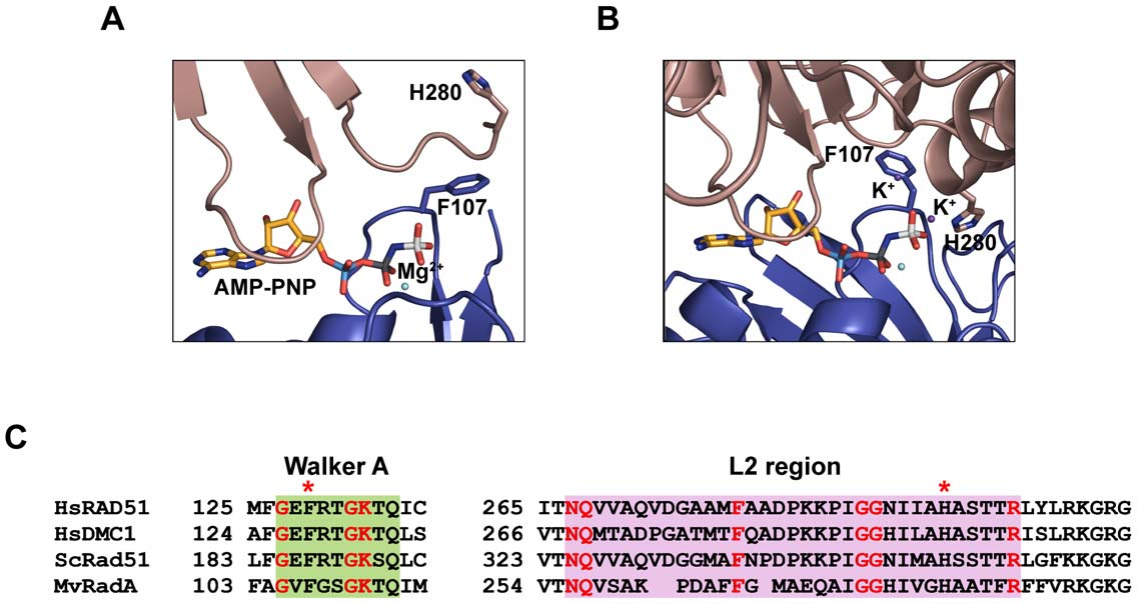
Cation-induced conformational rearragement of conserved amino acid residues of RadA. (**A**) Subunit interface of MvRadA (MvRAD51) structure in the absence of potassium cation (PDB code 1T4G). (**B**) Subunit interface region of MvRAD51 structure in the presence of potassium cation (PDB code 1XU4). Structural figures were generated using Pymol. (**C**) Sequence alignment of WalkerA/P-loop and L2 ssDNA binding region of *H. sapiens* (Hs), *S. cerevisiae* (Sc) and *M. voltae* (Mv) recombinases. HsRAD51 residues F129 and H294 are indicated with asterisks.

Despite the functional conservation with bacterial RecA, human RAD51 (HsRAD51) possesses an essential cation salt requirement for efficient strand exchange *in vitro*. The most effective cation is ammonium (NH_4_^+^), which is unlikely to be physiologically significant. However, potassium (K^+^) also enhances HsRAD51 recombinase functions to a lesser extent [14], [15] and has been shown to induce single stranded DNA-HsRAD51 complexes that structurally mimic active RecA NPFs [14]. The effect of salt has been attributed to an induced preferential single stranded DNA (ssDNA) binding by RAD51 over double stranded DNA (dsDNA) [15]. However, crystallographic analysis of the *methanococcus voltae* RadA (MvRAD51) has revealed important K^+^-induced amino acid and structural rearrangements within the intersubunit region of the NPF (Figure 1A and 1B). For example, the MvRAD51(F107) residue in the highly conserved Walker A box rotates away from the ATP binding interface to accommodate K^+^ cations (Figure 1b; [16]. This rotation of MvRAD51(F107) induces ordering of the L2 ssDNA binding domain and a conformational transition of MvRAD51(H280) that results in the formation of a direct hydrogen bond with the γ-phosphate group of the ATP analogue; which is suggested to be involved in polarizing the water molecule involved in ATP hydrolysis. These two residues are conserved in RAD51 homologs from archaebacteria to human (Figure 1C).

Here, we have examined substitution mutations of the analogous HsRAD51 residues to MvRAD51(F107) and MvRAD51(H280), HsRAD51(F129) and HsRAD51(H294). We find that HsRAD51(F129V) and HsRAD51(H294V) affect ATP hydrolysis (ATPase) activity but not adenosine nucleotide binding or ADP→ATP exchange. Moreover, they alter DNA binding properties in the presence of ATP and salt cations (K^+^ or NH_4_^+^) that ultimately results in defective recombinase functions. These data further delineate the importance of salt-induced allosteric changes at the subunit interface of HsRAD51 that promotes the formation of a functional NPF.

## Results

### Mutation of HsRAD51 inter-subunit residues F129 and H294 affect ATP turnover

**Figure 2:**
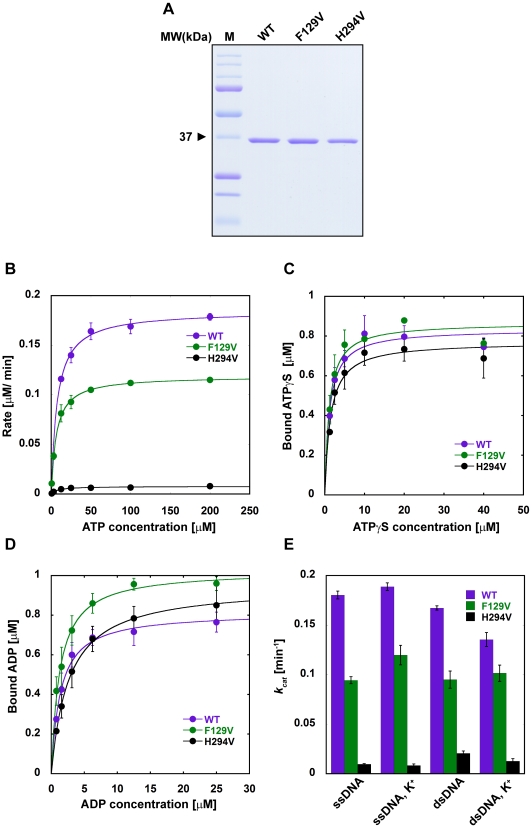
Mutation of HsRAD51(F129) and HsRAD51(H294) residues affect ATP turnover. (**A**) Purification of *wild type* and HsRAD51 substitution mutant proteins. Protein (1 µg) analyzed by 12% SDS-PAGE. (**B**) Steady-state ATPase activity with ssDNA in the presence of 150 mM KCl. (**C**) ATPγS binding by *wild type* and HsRAD51 substitution mutant proteins in the presence of ssDNA and 150 mM KCl. (**D**) ADP binding by *wild type* and HsRAD51 substitution mutant proteins in the presence of ssDNA and 150 mM KCl. (**E**) ATP turnover (*k_cat_*) with ssDNA and dsDNA in the presence and absence of KCl (K^+^). *k_cat_* values were calculated by Michaelis-Menten analysis. Error bars indicate standard deviation from at least three independent experiments.

HsRAD51(F129) and HsRAD51(H294) were mutated to Val (V) to minimize initial structural perturbations. The respective proteins were then purified to near homogeneity (Fig. 2A). The RecA/RAD51 family of proteins exhibits DNA dependent ATPase activity [17],[18],[19],[20],[21]. HsRAD51 possesses a modestly higher ATP turnover (k_cat_) in the presence of ssDNA compared to dsDNA, although it is about 150 times slower than the bacterial RecA [17], [19], [20], [21]. The HsRAD51(F129V) substitution mutation displays an ∼2-fold reduction in k_cat_ compared to the *wild type* HsRAD51, while the HsRAD51(H294V) substitution mutation nearly abolishes the ATPase activity (Fig. 2B, Table 1).

**Table 1:** Summary of ATP hydrolysis and nucleotide binding data of HsRAD51 *wild type* and HsRAD51(F129V) and HsRAD51(H294V) mutant proteins.

Kinetic Parameter		WT	F129V	H294V
***k_cat_*** ** (min^−1^)**	ssDNA	0.180±0.004	0.094±0.004	0.010±0.001
	ssDNA, KCl	0.190±0.004	0.119±0.009	0.008±0.001
	dsDNA	0.167±0.002	0.095±0.009	0.021±0.002
	dsDNA, KCl	0.135±0.007	0.101±0.008	0.013±0.002
***K_m_*** ** (µM)**	ssDNA	17.48±3.25	6.68±1.39	2.59±0.10
	ssDNA, KCl	9.34±1.42	6.40±0.032	6.40±3.31
	dsDNA	6.54±0.67	4.23±0.38	5.38±0.50
	dsDNA, KCl	5.52±0.26	4.90±0.24	8.82±0.26
***K_D_*** ** (µM) ATPγS**	ssDNA	2.49±0.46	1.53±0.69	0.817±0.14
	ssDNA, KCl	1.18±0.22	1.15±0.55	1.40±0.31
***B_max_*** ** (µM) ATPγS**	ssDNA	1.02±0.02	0.85±0.03	0.63±0.01
	ssDNA, KCl	0.83±0.04	0.87±0.04	0.77±0.03
***K_D_*** ** (µM) ADP**	ssDNA, KCl	1.24±0.32	1.27±0.11	2.66±0.10
***B_max_*** ** (µM) ADP**	ssDNA, KCl	0.82±0.05	1.03±0.02	0.95±0.01

Reduced k_cat_ could be due to several factors: an ATP binding deficiency, an ADP release deficiency or a deficiency in the catalytic step. To rule out the first two possibilities, we examined ATP binding, ADP binding and ADP-ATP exchange of the mutant proteins. We found no significant difference in ATP binding (Fig. 2C, Table 1), ADP binding (Fig. 2D, Table 1) or ADP-ATP exchange (data not shown). Previous studies have confirmed a small but reproducible salt induced ATPase catalytic rate enhancement in the presence of ssDNA compared to dsDNA [15]. Only RAD51(F129V) displayed a similar salt (150 mM KCl) induced catalytic rate enhancement in the presence of ssDNA (Fig. 2E). Together these observations are consistent with the conclusion that HsRAD51(H294) residue and to a significantly lesser extent the HsRAD51(F129) residue are required for appropriate ATP catalysis.

### HsRAD51(F129V) and HsRAD51(H294V) are deficient in D-loop formation and strand exchange

**Figure 3:**
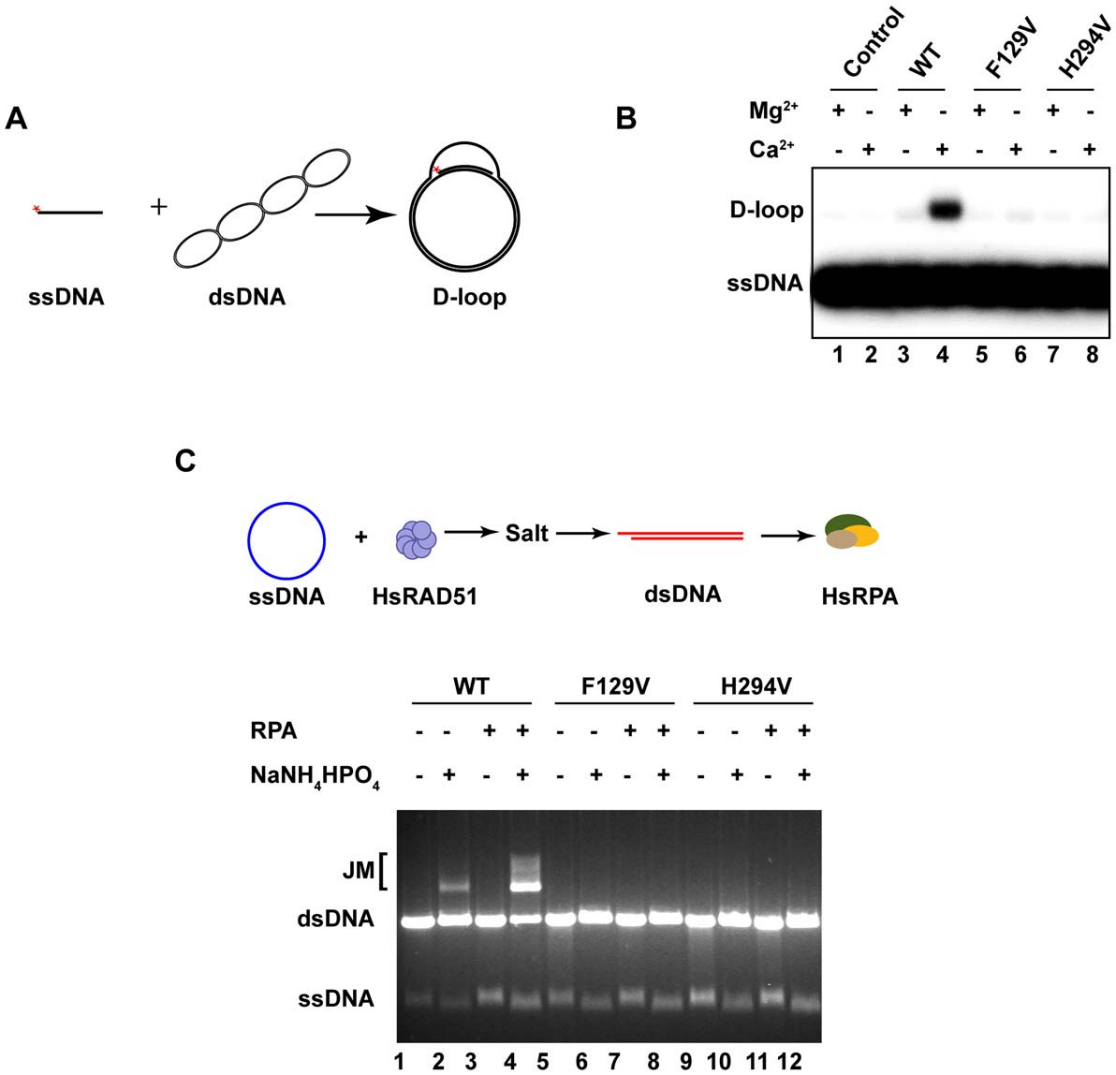
HsRAD51(F129V) and HsRAD51(H294V) are deficient in D-loop formation and strand exchange. (**A**) *In vitro* D-loop assay reaction schematic. (**B**) 0.8 µM of HsRAD51, HsRAD51(F129V), or HsRAD51(H294V) and [P^32^]-labeled ssDNA (90mer; 2.4 µM nt) were preincubated for 10 min at 37°C in the reaction buffer containing 1 mM ATP and 1 mM MgCl_2_ or CaCl_2_. Reactions were initiated by the addition of supercoiled pBS SK(-) plasmid DNA (35 µM bp). After 15 min, reactions were terminated by the addition of proteinase-K and SDS. Joint molecules (JMs) were analyzed on a 0.9% agarose gel. (**C**) Analysis of salt and RPA requirement for strand exchange. Reaction schematic shown above: HsRAD51 (5 µM) and φX174 circular ssDNA (30 µM nt) were pre-incubated with 2.5 mM ATP and 1 mM MgCl_2_ at 37°C for 5 m prior to the addition of 150 mM NaNH_4_HPO_4_ (if indicated) and linear φX174 dsDNA (15 µM bp). After 5 m, HsRPA (2 µM) was added (if indicated) and the incubation was continued for 3 h. Samples were deproteinized and analyzed on 0.9% agarose gel with 0.1 µg/mL ethidium bromide.

We examined the recombinase activity of the HsRAD51(F129V) and HsRAD51(H294V) substitution mutations. HsRAD51 catalyzes D-loop formation *in vitro* between a ^32^P-labeled 90-mer and a homologous supercoiled plasmid (Fig. 3A). Efficient D-loop formation occurs when ATP is present in a slow-hydrolysable state (e.g. in the presence of calcium) or when non-hydrolysable ATP analogues such as AMP-PNP are substituted for ATP [22], [23]. Importantly, the Walker A box mutant HsRAD51(K133R) that binds ATP normally but is defective in ATP hydrolysis, catalyzes efficient D-loop formation with ATP and Mg^2+^ [23]. These results have suggested that D-loop catalysis requires HsRAD51 to form and maintain an active ATP-bound NPF [22], [23], [24], [25].

In the presence of ATP, HsRAD51 catalyzed D-loop formation with Ca^2+^ but not with Mg^2+^ (Fig. 3B). Previous work has demonstrated that Ca^2+^ induces the formation of a stable ATP-bound NPF while Mg^2+^ allows the hydrolysis of ATP that results in a mixed (ADP/ATP) NPF that is largely inactive [22]. HsRAD51(F129V) and HsRAD51(H294V) did not catalyze D-loop formation in either Mg^2+^ or Ca^2+^ (Fig. 3B). Thus, even though HsRAD51(H294V) is ATPase deficient, and both HsRAD51(F129V) and HsRAD51(H294V) display ATP binding that is comparable to the *wild type*, these mutant proteins are incapable of catalyzing D-loop formation. Collectively these results indicate that suppression of ATP hydrolysis alone is not sufficient to confer enhanced D-loop recombinase activity.

Catalysis of strand exchange between a duplex DNA substrate and a homologous single stranded circular DNA substrate is a hallmark of RecA/RAD51 proteins [18], [24], [26], [27], [28], [29]. Unlike D-loop formation that occurs in low salt, strand exchange requires specific cations (either NH_4_^+^ or K^+^) to activate HsRAD51 activity [14], [15], [30]. Using φX174 virion DNA and ApaL1 linearized φX174 dsDNA we compared the strand exchange activity of the HsRAD51(F129V) and HsRAD51(H294V) mutant proteins to *wild type* HsRAD51. We also examined the stimulatory effect of the human single-stranded binding complex RPA (HsRPA). Neither HsRAD51(F129V) nor HsRAD51(H294V) were able to form joint molecules in the presence of salt and/or RPA (Fig. 3C). We can conclude that the HsRAD51(F129) and HsRAD51(H294) residues are critical for cation-induced HsRAD51 recombinase functions.

### HsRAD51(F129) and HsRAD51(H294) are essential for ATP-dependent DNA binding

**Figure 4:**
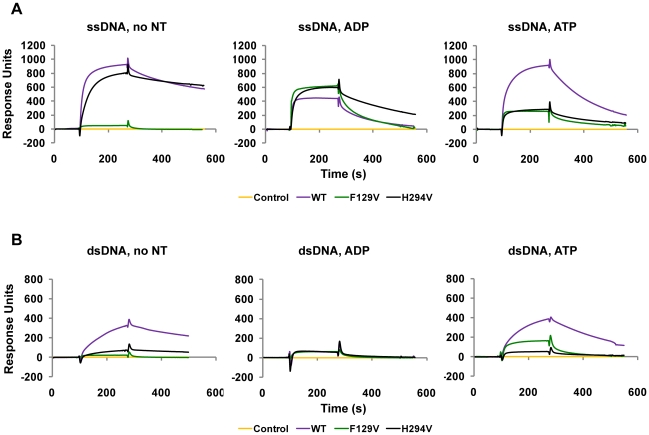
F129 and H294 of HsRAD51 are critical for DNA binding in the presence of ATP. (**A**) ssDNA binding analysis of *wild type* and HsRAD51 substitution mutant proteins by surface Plasmon resonance (SPR, Biacore) in the absence of an adenine nucleotide, in the presence of ADP and in the presence of ATP. Association and dissociation curve corresponding to 800 nM of each protein is shown. (**B**) dsDNA binding analysis of *wild type* and HsRAD51 substitution mutant proteins.

We examined real-time HsRAD51 ssDNA and dsDNA binding by surface plasmon resonance (SPR, Biacore). Biotinylated dT_50_ ssDNA and 50 bp dsDNA were immobilized on a streptavidin-coated flow-cell surface. SPR measures the change in the refractive index that reflects protein binding and/or dissociation from DNA. We analyzed ssDNA and dsDNA binding in salt conditions similar to those used in for strand exchange. We titrated the protein from 100 nM to 1.6 µM in the presence of ADP, ATP and in the absence of adenosine nucleotide. For clarity the binding and dissociation curves shown correspond to 800 nM of protein (Fig. 4). After protein injection nucleoprotein filament formation was analyzed for 150 sec. For steady-state protein disassembly, protein free buffer was injected into the flow cells for 300 sec. Because the ssDNA and dsDNA were introduced into separate channels we could simultaneously examine protein binding and dissociation to the two DNA substrates. In the absence of a adenosine nucleotide only the *wild type* HsRAD51 and HsRAD51(H294V) bound to ssDNA (Fig. 4A, left panel, Table 2). In the presence of ADP, the *wild type* and mutant variants bound to ssDNA, but dissociated rapidly (Fig. 4A, middle panel, Table 2). These results are consistent with previous studies that have suggested protein turnover associated with ADP-bound RAD51 [22], [31]. Interestingly, in the presence of ATP, *wild type* HsRAD51 bound ssDNA while both HsRAD51(F129V) and HsRAD51(H294V) were largely defective in ssDNA binding (Fig. 4A, right panel, Table 2). Only *wild type* HsRAD51 displayed dsDNA binding in the presence of ATP as well as in the absence of a nucleotide (Fig. 4B, Table 2). These results suggest that the HsRAD51(F129) and HsRAD51(H294) residues are important for the ATP induced DNA binding that ultimately results in an active NPF required for recombinase function.

**Table 2:** Summary of DNA binding data of HsRAD51 *wild type* and HsRAD51(F129V) and HsRAD51(H294V) mutant proteins.*

DNA binding parameter		WT	F129V	H294V
***k_on_*** ** (nM^−1^ s^−1^)×10^−6^**	ssDNA,no NT	111	>150	94
	ssDNA, ADP	469	218	94
	ssDNA, ATP	59	293	114
	dsDNA,no NT	7	>40	4
	dsDNA, ADP	>70	59	>90
	dsDNA, ATP	14	36	>10
***k_off_*** ** (s^−1^)×10^−3^**	ssDNA,no NT	1.7	>50	2.6
	ssDNA, ADP	9.5	11.7	4.1
	ssDNA, ATP	5.3	17.7	5.4
	dsDNA,no NT	1.9	>20	1.9
	dsDNA, ADP	>10	26.2	>10
	dsDNA, ATP	3.7	18.2	>30
***K_D_*** ** (nM)**	ssDNA,no NT	15	NA	28
	ssDNA, ADP	20	54	43
	ssDNA, ATP	90	60	47
	dsDNA,no NT	264	NA	472
	dsDNA, ADP	NA	446	NA
	dsDNA, ATP	262	508	NA

* In some cases, binding and/or dissociation occurs very rapidly, and cannot be accurately determined by SPR. For these cases we simply report a lower bound for *k_on_* and *k_off_*, and cannot determine an accurate dissociation constant, *K_D_* (Indicated by NA).

The abnormal DNA binding did not appear to fully account for the altered steady-state ATPase activity of HsRAD51(F129V) and HsRAD51(H294V) (compare Fig. 2E, Table 1 with Fig. 4, Table 2). This is particularly true for HsRAD51(H294V) which showed reduced but not absent binding to ssDNA, yet near background ATPase activity. The dissociation of ssDNA binding activity from ATPase activity suggests that some other function(s) of these proteins are compromised. Based on the location of these residues in the crystal structures we postulate that cation induced conformational transitions are defective in HsRAD51(F129V) and HsRAD51(H294V) (see Figs. 1A and 1B). These conformational transitions do not influence ADP/ATP binding or ADP→ATP exchange, but do affect the ability of the mutant proteins to interact appropriately with DNA and to properly catalyze ATP hydrolysis.

## Discussion

Structural studies of the MvRAD51 and *S.cerevisiae* RAD51 (ScRAD51) have detailed significant conformational transitions associated with the NPF. The ScRAD51 residues analogous to HsRAD51(F129) and HsRAD51(H294), ScRAD51(F187) and ScRAD51(H352), were found in two alternate conformations within the NPF [32], [33]. In one conformation ScRAD51(H352) was positioned above the ATP binding site while in the other ScRAD51(F187) excluded ScRAD51(H352) by moving it out of the active site. Importantly, K^+^ cations induce similar conformational transitions of MvRAD51 of the analogous residues to HsRAD51(F129) and HsRAD51(H294), MvRAD51(F107) and MvRAD51(H280). In the presence of K^+^ the MvRAD51(F107) rotates away from the ATP catalytic interface and MvRAD51(H280) rotates in to form a hydrogen bond interaction with the γ-phosphate of ATP, while at the same time ordering the L2 ssDNA binding domain [16].

Our studies support the importance of these conformational transitions. While MvRAD51(F107V) can bind and hydrolyze ATP, it displays significantly reduced ssDNA binding in the presence of ATP. These results suggest an impaired ability to incite the conformational transitions necessary to the form an active NPF on ssDNA. We speculate that this impairment involves an inability of the smaller Val residue to incite ordering of the L2 ssDNA-binding domain that is normally provoked by cation-induced rotation of the Phe residue away from the ATP catalytic interface. In contrast, HsRAD51(H294V) binds but does not hydrolyze ATP, binds ssDNA in the absence of adenosine nucleotide, yet displays significantly reduced ssDNA binding in the presence of ATP. We speculate that these impairments are the result of an inability of the Val residue to form an appropriate hydrogen bond interaction with the γ-phosphate of ATP in spite of the fact that L2 ssDNA-binding domain ordering has been provoked by the MvRAD51(F107) residue [34]. Modeling of the HsRAD51 structure strongly support such an allosteric communication between ATPase site and L2 ssDNA binding region [35], [36]. A recent mutational analysis also revealed that the subunit interface residue ScRAD51(H352) is critical for functional NPF formation and strand exchange activity [37]. Ultimately, the defects associated with both HsRAD51(F129V) and HsRAD51(H294V) result in defective D-loop and strand exchange functions. Because both proteins bind ADP and ATP similar to the *wild type* HsRAD51, the functional defects are unlikely to be a result of improper folding and/or aggregation.

For RecA/RAD51 proteins ATP binding but not necessarily hydrolysis, is sufficient to catalyze strand exchange [23], [26], [27], [28], [38], [39]. It has been suggested that RAD51 might utilize binding of ATP and the resulting release of the hydrolysis product as a conformational switch for regulating recombinase function similar to other members of the AAA+ superfamily [40]. Our data is consistent with the hypothesis that the HsRAD51(F129) and HsRAD51(H294) residues along with salt cations may play a significant role in this conformational switch during HRR.

## Materials and Methods

### HsRAD51 Protein Expression and Purification

The mutant RAD51 genes F129V and H294V were constructed using wild type HsRAD51 gene in the pET24d vector system (Novagen) using conventional PCR. All mutations were confirmed by DNA sequencing. Wild type and mutant hRAD51 proteins were expressed and purified following previously published protocols [17], [41]. Briefly, hRAD51 was expressed in *E. coli* BLR strain and precipitated using spermidine-HCl. Resuspended pellet was purified using Reactive-Blue-4-agarose, Heparin sepharose, hydroxyapatite and Mono Q column chromatography. Purity of the fractions was verified by SDS-PAGE analysis. HsRPA was expressed in BL21(AI) cells using pET11d-tRPA purified as described [42], except for the resuspension of cells where HI buffer was supplemented with 100 mM KCl.

### DNA Substrates

φX174 single-stranded (ss) virion DNA and replicative form I (RFI) were purchased from NEB. φX174 RFIII was obtained by linearizing RFI with ApaLI restriction enzyme and gel purifying with Qiaquick Gel Extraction kit (Qiagen). For surface plasmon resonance (SPR) analysis, a 5′ biotinylated oligo dT_50_ was used as ssDNA and for dsDNA 5′ biotinylated 50-mer 5′-TCG AGA GGG TAA ACC ACA- ATT ATT GAT ATA AAA TAG TTT TGG GTA GGC GA was annealed with its complement. D-loop assay substrates were prepared as described [43].

### ATPase assay

ATP hydrolysis was measured as previously described [17]. Reactions were performed in 10 µL volumes in Buffer A containing 20 mM HEPES (pH 7.5), 10% glycerol, 100 µg/mL BSA, 1 mM DTT and 1 mM MgCl_2_ and when indicated, 150 mM KCl. Each reaction mixture contained RAD51 (1 µM) and 6 µM (nt or bp) of φX174 ssDNA or dsDNA. Reactions were initiated by the addition of protein and incubation at 37°C. After 1 hr 400 µL of 10% activated charcoal supplemented with 10 mM EDTA was added to terminate the reaction and incubated on ice for another 2 hrs. After centrifuging for 10 min 50 µL duplicate aliquots were taken for counting [^32^P] free phosphate by Cerenkov method. Kinetic parameters were obtained by fitting data into Michaelis-Menten equation using the software Kaleidagraph (Synergy software).

### ATPγS/ADP binding assay

Experimental procedure was as previously described [17]. Reactions were performed in Buffer A and 150 mM KCl, when indicated. RAD51 (1 µM) and 6 µM (nt) φX174 ssDNA were used with the indicated amount of [γ-^35^S]ATPγS or [^3^H]ADP. After a 30 min incubation at 37°C, reactions were kept on ice until filtered. Reaction was added into 4 mL of ice-cold reaction buffer and filtered through HAWP nitrocellulose filters (Millipore) presoaked in the same buffer. Another 4 mL of buffer was used to wash the membrane and the filter was dried for 2 hrs. Radioactivity was counted after an overnight incubation in liquid scintillation fluid.

### ADP-ATP exchange assay

Reactions were performed in Buffer A with 150 mM KCl. RAD51(1 µM) and 6 µM (nt) φX174 ssDNA was incubated with 3 µM of [^3^H]ADP in a 60 µL reaction volume at 37°C for 10 mins. ADP to ATP exchange was initiated by addition of cold ATP to a 5 mM final concentration. 10 µL aliquots were withdrawn at indicated time points, filtered and analyzed as in ADP binding.

### SPR analysis

Biotinylated DNA was immobilized on a streptavidin-coated chip (GE) for the analysis. Protein binding and dissociation were analyzed at 25°C with a 5 µL/min flow rate on a Biacore 3000 (GE). Reactions were performed in Buffer containing 20 mM HEPES (pH 7.5), 10% glycerol, 1 mM DTT and 1 mM MgCl_2_, 0.005% surfactant P-20 (GE), 2.5 mM of the indicated nucleotide and 150 mM KCl when indicated. For experiments with Ca^2+^, 1 mM CaCl_2_ was used instead of MgCl_2_. DNA binding was determined by injecting 100 nM, 200 nM, 400 nM, 800 nM and 1.6 µM of protein simultaneously into ssDNA and dsDNA containing flow channels to ensure saturated binding.

### D-loop assay

2.4 µM (nt) labeled 90-mer was incubated with HsRAD51 (0.8 µM) in buffer A with 1 mM ATP supplemented with the indicated amounts of MgCl_2_ or CaCl_2_ at 37°C for 10 min. Reaction was initiated by adding 35 µM (bp) supercoiled pBS-SK(-) plasmid and incubated further for 15 min. Samples were deproteinized by addition of 1% SDS and 1 mg/mL proteinase K to a final concentration and incubated for 15 min at 37°C, mixed with 1/5 volume of gel loading dye and analyzed on 0.9% agarose gel in TAE buffer, run at 4 V/cm at 25°C. D-loops were quantified after drying and exposure to PhosphoImager screens.

### DNA Strand Exchange Assay

HsRAD51 (5 µM) and 30 µM (nt) φX174 circular ssDNA were pre-incubated in buffer containing 20 mM HEPES (pH 7.5), 10% Glycerol, 1 mM DTT, 1 mM MgCl_2_ supplemented with 2.5 mM of ATP at 37°C for 5 min before addition of 150 mM of the indicated salt and 15 µM (bp) linear φX174 dsDNA. After another 5 min incubation 2 µM of HsRPA was added and the incubation was continued. After 3 hrs samples were deproteinized by addition of 3 µL of stop buffer containing 2% SDS and 10 mg/mL proteinase K, and analyzed on 0.9% agarose gel in TAE buffer. Electrophoresis was carried out at 4 V/cm at 25°C with 0.1 µg/mL ethidium bromide.
